# Supplementary material for: Is Lipid Specificity Key to the Potential Antiviral Activity of Mouthwash Reagent Chlorhexidine against SARS-CoV-2?
Source: Membranes (Basel). 2022 Jun 14;12(6):616. doi: 10.3390/membranes12060616 (PMC9230368; doi:10.3390/membranes12060616)
Supplement: Supplementary file 1 [file membranes-12-00616-s001.zip › membranes-1729837-supplementary.pdf]

Supporting Information

Is Lipid Specificity Key to the Potential Antiviral Activity of Mouthwash Reagent Chlorhexidine against SARS-CoV-2?

Arun K. Rathod [1], Chetan S. Poojari [2,*] and Moutusi Manna [1,*]

[1] Applied Phycology and Biotechnology Division, CSIR Central Salt & Marine Chemicals Research Institute, Bhavnagar 364002, Gujarat, India; rathod.csmcri21j@acsir.res.in
[2] Theoretical Physics and Center for Biophysics, Saarland University, 66123 Saarbrücken, Germany
* Correspondence: chetan.poojari@uni-saarland.de (C.S.P.); mmanna@csmcri.res.in (M.M.); Tel.: +49-1628779734 (C.S.P.); +91-278-2567760 (M.M.)

Table S1. Physical bilayer properties.

Properties	VM	CHX in VM			PM	CHX in PM		
Area per lipid (APL)	61.51 ± 0.01	62.77 ± 0.01	62.81 ± 0.01	62.73 ± 0.01	43.33 ± 0.007	44.27 ± 0.005	44.42 ± 0.006	44.27 ± 0.005
ΔAPL	0.00	1.26	1.30	1.22	0.00	0.94	1.09	0.94
Bilayer thickness (D_{P-P})	40.65 ± 0.008	40.19 ± 0.005	40.15 ± 0.005	40.21 ± 0.005	47.22 ± 0.006	46.61 ± 0.004	46.49 ± 0.005	46.62 ± 0.004
ΔD_{P-P}	0	-0.46	-0.5	-0.44	0	-0.61	-0.73	0.60

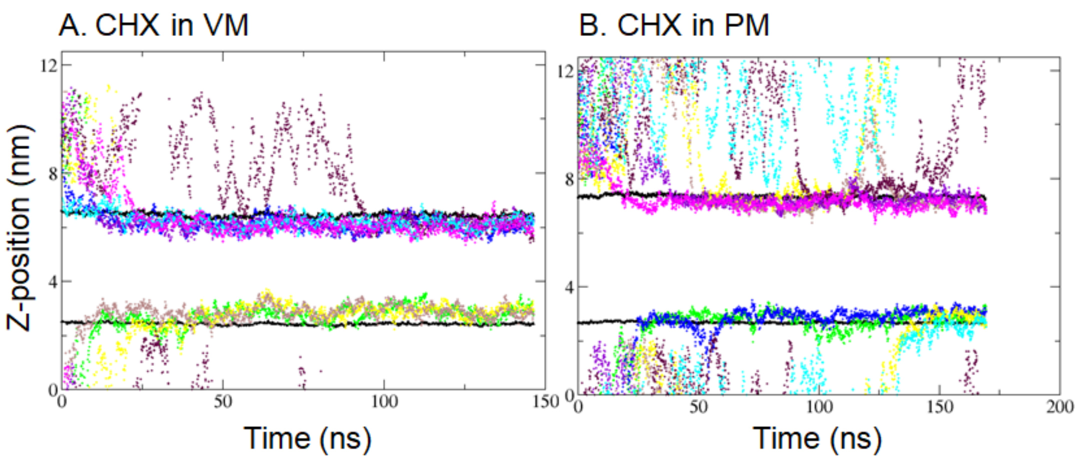

Figure S1. The center of mass distribution of individual CHX molecules along bilayer normal, starting from CHX in water. The black lines represent the average planes of P atoms of two bilayer leaflets. Individual CHX molecules are colored separately.

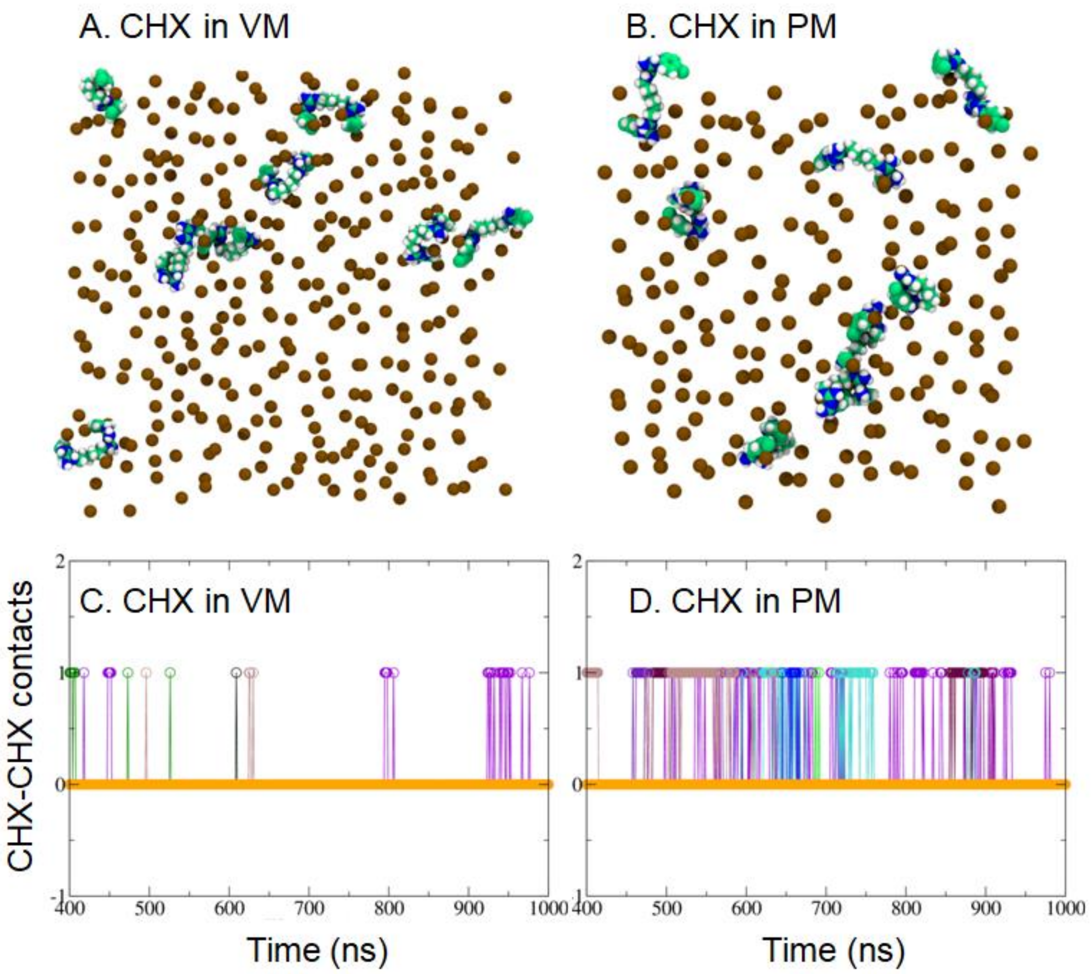

Figure S2. MD simulation snapshots represent the top view of CHX binding to (A) VM and (B) PM membranes. The membrane are represented by lipid P atoms which are rendered as brown spheres. Ions and water are not shown for clarity. (C-D) Time profile of CHX-CHX contacts. A contact is defined when non-hydrogen atoms of two CHX molecules come at distance ≤ 0.5 nm of each other. In the figures, 1 represents that two CHX are in contact and 0 stands for not in contact.

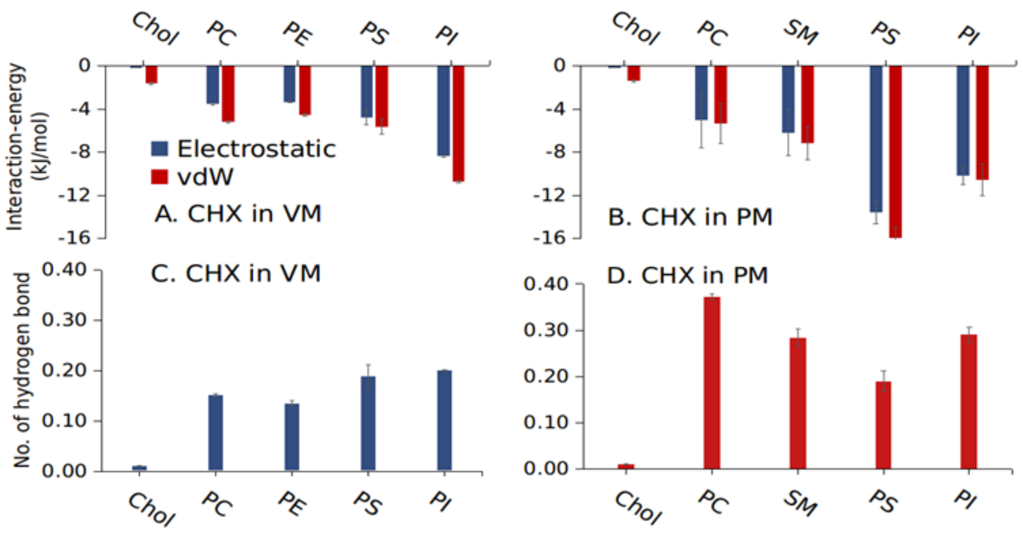

Figure S3. Interaction energies and number of hydrogen bonds of CHX per lipid headgroup types.

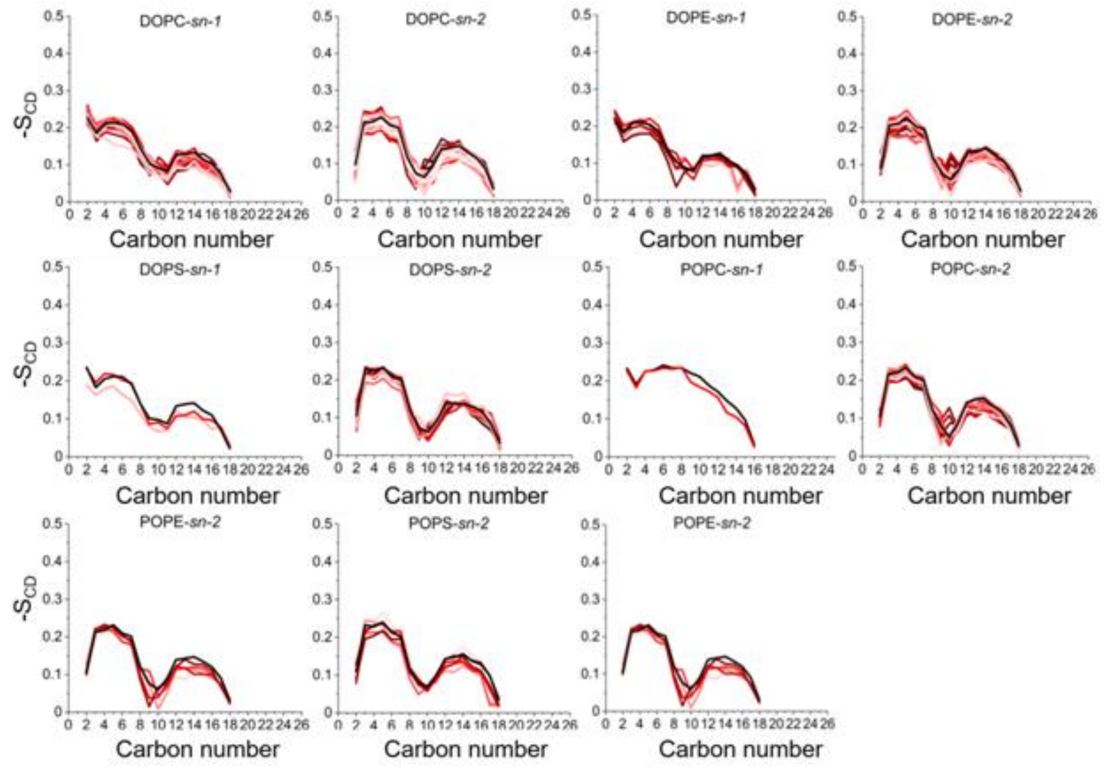

Figure S4. Order parameters (-S_{CD}) profiles of individual lipids bound to CHX in the viral model membrane. The order parameter profile from the pure VM membrane without CHX is shown as black lines.

The order parameter of lipid acyl chain, S_{CD}, is calculated using the following equation:

$$S_{CD} = 1/2 \langle 3\cos^2(\theta_z) - 1 \rangle$$

where θ is the angle of a C−D vector with respect to the bilayer normal.

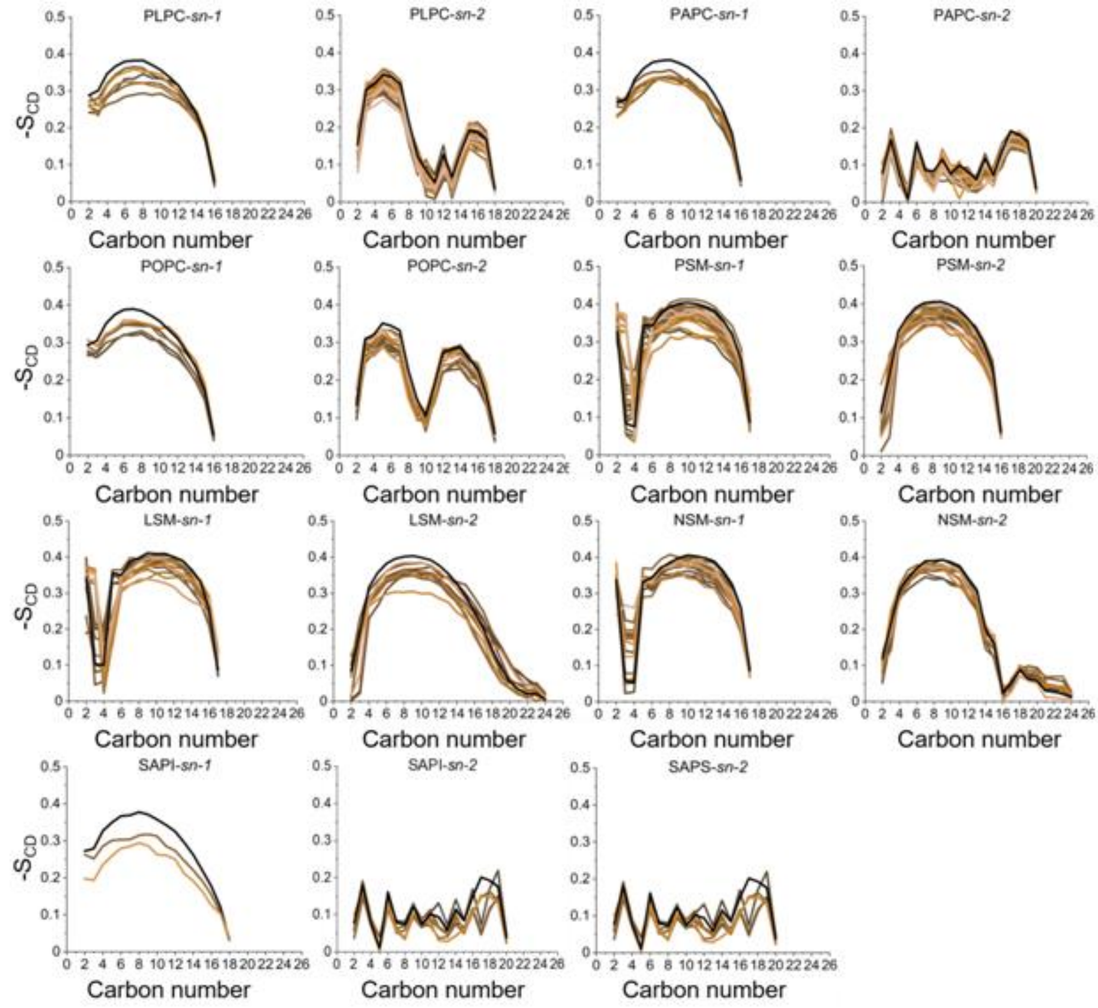

Figure S5. Order parameters (-S_{CD}) profiles of individual lipids bound to CHX in the plasma model membrane. The order parameter profile from the pure PM membrane without CHX is shown as black lines.
